# Hyponatraemia and seizures in Merrem’s hump-nosed pit viper (*Hypnale hypnale*) envenoming: a case report

**DOI:** 10.1186/s13256-018-1756-2

**Published:** 2018-08-02

**Authors:** Umesh de Silva, Chamara Sarathchandra, Hemal Senanayake, Senaka Pilapitiya, Sisira Siribaddana, Anjana Silva

**Affiliations:** 1 Professorial Medical Unit, Teaching Hospital, Anuradhapura, 50000 Sri Lanka; 2 Department of Medicine, Faculty of Medicine and Allied Sciences, Rajarata University of Sri Lanka, Saliyapura, 50008 Sri Lanka; 3 Department of Parasitology, Faculty of Medicine and Allied Sciences, Rajarata University of Sri Lanka, Saliyapura, 50008 Sri Lanka

**Keywords:** *Hypnale hypnale*, Envenoming, Hyponatremia, Generalized tonic-clonic seizures

## Abstract

**Background:**

Hump-nosed pit vipers (Genus: *Hypnale*) are medically important snakes in Sri Lanka and South India. Merrem’s Hump-nosed pit viper (*Hypnale hypnale*) frequently leads to potentially fatal envenomings in Sri Lanka and India. Venom-induced consumption coagulopathay (VICC), local envenoming and acute kidney injury (AKI) are the commonest effects of the envenoming by this snake.

**Case presentation:**

We report a previously unreported presentation of *H. hypnale* envenoming, with an isolated urinary salt loss leading to moderate hyponatraemia resulting seizures. The patient was treated with careful fluid and electrolyte management. No antivenom is currently available for *H. hypnale* envenoming.

**Conclusion:**

In the absence of any evidence of venom induced consumptive coagulopathy, acute kidney injury and cerebral haemorrhage, we hypothesize that this effect is likely due to the presence of a natriuretic peptide in *H. hypnale* venom, similar to the natriuretic peptides identified in few other snake venoms.

## Background

Hump-nosed pit vipers (genus: *Hypnale,* sub-family: Crotalinae) are medically important snakes in Sri Lanka and South India [1]. Of the three species of this genus, Merrem’s hump-nosed pit viper (*Hypnale hypnale*) has increasingly been recognized as a snake with high medical importance in the region, due to frequent and potentially fatal envenomings [2]. In Sri Lanka, envenoming by *H. hypnale* accounts for 35–45% of the hospital admissions due to snakebites [3]. *H. hypnale* is known to cause severe local envenoming, VICC and AKI [2, 4]. In addition, thrombotic events causing cerebral and myocardial ischemia, and, renal tubular acidosis leading to hyperkalemia have rarely been reported [5]. Here we report previously unreported presentation of *H. hypnale* envenoming leading to hyponatremia, hypovolemia, urinary salt loss and generalize tonic-clonic seizures, with the absence of any other systemic effects of envenoming.

## Case presentation

A previously well, 64-year-old South Asian woman who was not on any medication presented to the Teaching Hospital, Anuradhapura, Sri Lanka, with a history of snake bite, five hours before admission while gardening. The offending snake specimen was brought to the hospital and was identified as a Merrem’s hump-nosed viper (*Hypnale hypnale* - the only species of Hump-nosed pit viper that exists in the area), by the doctor at the hospital emergency department. The patient complained of mild swelling and pain at the bite site and epigastric pain. She was fully conscious, alert and oriented and was not under influence of any substance or alcohol. The only first-aid the patient had received was washing of the bite site. There was mild swelling and tenderness of the right foot with two fang marks below the right lateral malleolus. Her heart rate was 106 beats per minute, blood pressure was 95/60 mmHg on supine position. The patient had mild postural dizziness and, also complained of increased thirst and appeared dehydrated. The twenty-minute whole blood clotting test (WBCT20) was < 20 min and the International Normalized Ratio (INR) was 1.05. She was kept under observation and, was not given antivenom as usual because the only available antivenom (Indian Polyvalent antivenom) is not raised against Hump-nosed pit vipers. The rest of her physical examination was unremarkable. Oral fluids intake of 0.6 L and intravenous infusion of 0.9% saline in the rate of 100 ml/hour (total of 3 L over 24 hours) was commenced. The patient received tetanus toxoid and oral cloaxacillin 500 mg 6 hourly.

Next day, 23 hours after the bite, she developed an episode of generalized tonic-clonic seizure which lasted 30 minutes, followed by two episodes of similar seizures two and four hours after the initial episode. Her serum electrolytes following the first seizure showed profound hyponatremia (Na^+^ 118 mEq/L; normal: 135–146 mEq/L) and normal serum potassium (3.8 meq/L; normal 3.5–5 mmol/L). The plasma glucose at the time of the seizure was 152 mg/dl. WBCT20 and INR were normal. Her blood cell count and hemoglobin concentration were normal. The serum creatinine was 0.81 mg/L and the blood urea nitrogen level was 2.3 g/L. The serum corrected calcium was normal (2.46 mmol/L; normal: 2.1–2.6 mmol/l). Serum osmolality was low (257 mosm/kg water; normal: 280–300 mosm/kg water). Urine biochemistry showed high urinary sodium of 164 meq/L (normal: 20 meq/L), increased fractional sodium excretion 12.76% (normal < 1%), low urinary potassium 20.4 meq/L (normal: 25–100 meq/24 hour) and high fractional potassium excretion 52.14% (normal < 10%). She had no past history of any co-morbidities. She had no history of seizures and was not on any medications. The ultrasound scan showed normal kidneys. Computer Tomography, Magnetic Resonance Imaging of the brain, cerebral venogram and electroencephalogram were all normal. She was treated with oxygen (6 L/min) via a face mask, intravenous diazepam, oral sodium valproate and the hydration was maintained with 0.9% saline infusion and additional oral fluids to a total 3 L per day after first 24 hours with close monitoring of vital signs and fluid balance. The renal salt loss and the clinical and biochemical parameters improved after careful volume replacement with 0.9% saline for five days. She was discharged from the ward seven days after the snake bite. Her serum sodium level (140 mEq/L) and the urinary osmolality on discharge was normal. Although it was planned to review her after four weeks, the patient defaulted from follow up.

## Discussion and conclusions

In this patient, *H. hypnale* envenoming resulted in urinary salt loss, profound hyponatraemia and hypovolemia. Hyponatraemia was the most likely cause of the generalized tonic-clonic seizures. The patient only had mild local envenoming and had no evidence of VICC, haemorrhage or AKI.

A similar clinical picture has previously been observed in *Bungarus candidus* and *B. multicinctus* envenomings in Vietnam where hyponatraemia due to urinary salt loss was also reported, despite normal anti-diuretic hormone levels [6, 7]. Venoms of the following snakes are known to possess natriuretic peptides: Green Mambas (*Dendroaspi sangusticeps*), Inland taipan (*Oxyuranus microlepidotus*), Brown snake (*Pseudonaja textillis*), Mulga snake (*Pseudechis australis*), Horned vipers (*Psudocerastus persicus*), South American Rattlesnake (*Crotalus durissus cascavella*) and Lebetine viper (*Macrovipera lebetina*). These peptides are similar to the endogenous natriuretic peptides like Atrial Natriuretic Peptide and the Brain Natriuretic Peptide [8]. Dendroaspis Natriuretic Peptide from the Green Mamba venom has been pharmacologically well-characterized [8]. Similar to the endogenous natriuretic peptides, the venom-derived natriuretic peptides can act on the kidneys to increase the glomerular filtration rate and decrease sodium and water resorption, leading to a urinary salt loss [8]. Therefore, it could be assumed that the present patient developed urinary salt loss due to the likely presence of a natriuretic peptide in the yet underexplored venom of *H. hypnale*.

Although yet unreported, a similar clinical picture could also be theoretically resulted in intracranial haemorrhage due to the VICC, that can potentially result in Cerebral Salt Wasting syndrome (CSW) [9]. However, our patient clearly had no VICC and had no clinical or radiological evidence of an intracranial haemorrhage, hence CSW is unlikely. Nevertheless, the management could be of no difference to CSW, with the correction of the fluid loss with a positive salt balance and by matching the urinary output with volume repletion [10]. In this case, the unavailability of an antivenom that covers Hump-nosed pit vipers has possibly led to the progression of the hyponatraemia.

This case illustrates an unexpected presentation of isolated urinary salt loss in *H. hypnale* envenoming, that resulted in profound hyponatraemia and seizures. The physicians should be aware of the rare presentations of the envenomings by common, medically important snakes.

## Abbreviations

AKI: Acute kidney injury
CSW: Cerebral salt wasting
INR: International normalized ratio
VICC: Venom-induced consumption coagulopathay
WBCT20: Twenty-minute whole blood clotting test
